# Fungal Infections in the Caribbean: A Review of the Literature to Date

**DOI:** 10.3390/jof9121177

**Published:** 2023-12-08

**Authors:** Nicole Gousy, Bharadwaj Adithya Sateesh, David W. Denning, Krystal Latchman, Edmond Mansoor, Jillwin Joseph, Prasanna Honnavar

**Affiliations:** 1 Department of Clinical Sciences, American University of Antigua College of Medicine, St. Johns 1451, Antigua and Barbuda; nicoleg@auamed.net; 2 Postdoctoral Teaching Intern, American University of Antigua College of Medicine, St. Johns 1451, Antigua and Barbuda; badithya@auamed.net (B.A.S.); klatchman@auamed.net (K.L.); 3 Manchester Fungal Infection Group, The University of Manchester and Manchester Academic Health Science Centre, CTF Building, Grafton Street, Manchester M13 9NT, UK; denning@manchester.ac.uk; 4 Department of Clinical Medicine, American University of Antigua College of Medicine, St. Johns 1451, Antigua and Barbuda; emansoor@auamed.net; 5 Department of Microbiology and Immunology, American University of Antigua College of Medicine, St. Johns 1451, Antigua and Barbuda; jjillwin@auamed.net

**Keywords:** fungi, infections, infectious disease, geographical, Caribbean, mycoses

## Abstract

The most common fungal infections reported from the Caribbean include dermatophytosis, candidiasis, pneumocystis, aspergillosis, histoplasmosis, and cryptococcosis. The Caribbean is hyperendemic for histoplasmosis, with high population exposures. Fungal infections are a significant public health problem in the Caribbean, with rates varying depending on the specific country or region. In Trinidad and Tobago, the fungal burden accounts for 3.3% of the 1.4 million population, while in Jamaica, with a population of 2.9 million, over 57,600 people suffer from fungal infections each year. A study in the Dominican Republic estimated that approximately 221,027 (2%) of over 10 million people have a serious fungal infection. Fungal infections accounts for 21.9% of all skin infections in Haiti. The diagnosis of fungal infections in the Caribbean can be challenging, as access to laboratory testing and specialized medical services is limited in many areas. Access to antifungal medications can also be a challenge in some areas, and antifungal resistance has been reported.

## 1. Introduction

Fungal infections are a significant public health concern worldwide, including in the Caribbean region. The Caribbean is a diverse region with a variety of environmental conditions, including tropical rainforests, coastal plains, and volcanic islands. These unique environmental factors can contribute to the growth and spread of various fungal species that can cause infections in humans, animals, and plants. The incidence of fungal infections is probably growing at a rapid rate, and this poses a titanic challenge to healthcare providers.

Fungal infections in humans range from mild to severe. Fungi causes five main categories of infections: namely, skin, hair, and nails (superficial), mucosal, allergic, chronic (including pulmonary and subcutaneous), and invasive [1]. Superficial and mucosal infections are not life-threatening and are more easily diagnosed and treated. Allergic fungal infection includes allergic fungal rhinosinusitis and complicating asthma, allergic bronchopulmonary aspergillosis (ABPA), and severe asthma with fungal sensitization (SAFS). In contrast, invasive infections are life-threatening and usually caused by an opportunistic organism affecting an immunocompromised host or by a dimorphic fungus endemic to a specific geographical area [1]. Overall, these infections can occur in both immunocompromised and immunocompetent individuals and lead to significant morbidity and mortality.

Fungal infections in humans are more prominent in the tropics and subtropics most likely due to their warm climate [2]. Species causing superficial infections such as *Malassezia*, *Hortaea*, *Trichosporon*, and *Piedraia* are seen altogether in the tropics and some coastal regions. Dermatophytes including the *Trichophyton*, the *Microsporum*, and the *Epidermophyton* are also found internationally in the tropics, apart from certain species, like the *T. schoenleinii*, which is virtually restricted to Iran, Africa, and China, and the *T. concentricum*, which is restricted to Polynesian and Melanesian archipelagos and Central and South America [2]. Subcutaneous mycoses include chromoblastomycoses (most common in India, Thailand, Madagascar, Amazonian regions, and the Dominican Republic), eumycetoma (more common in India and Africa) and sporotrichosis (found globally), and lobomycosis (found mostly in the Amazon rainforest) [2]. The Caribbean region has the perfect humid tropical climate for these fungi. Chronic pulmonary aspergillosis and histoplasmosis are found globally and are under-recognized. Invasive fungal infections are also found globally, affecting those infected with HIV/AIDS, leukemia, lung and other cancers, corticosteroid-treated patients, and those in intensive care. Fungal infections contribute to an increased burden on healthcare systems in the Caribbean, most of which have socio-economic issues as well.

Despite the likely high prevalence of fungal infections in the Caribbean, which includes more than 30 countries and territories, there is limited research on their epidemiology, clinical presentation, and management. A recent global summary of cryptococcal disease in HIV patients found no data from the Caribbean, for example, but, using central American data, estimated 2000 positive cases with 1700 meningitis cases and 1000 deaths [3]. Surveillance is not undertaken for any fungal infection in any Caribbean country. The literature on HIV is vast and only peripherally touches on fungal infections, and detailing all these mentions is not the purpose of this paper. This review aims to provide a comprehensive overview of fungal infections in the Caribbean. Understanding the epidemiology and opportunities for better management of fungal infections in the Caribbean is essential for the effective prevention and control of these infections and for improving the overall health of the region’s population. In discussing epidemiological data, it is crucial to acknowledge the inherent limitations associated with estimates, as they may tend to overstate the true incidence or prevalence of fungal diseases. This cautionary note serves to underscore the need for a nuanced interpretation of the presented figures and emphasizes the importance of considering potential overestimations in our examination of fungal disease trends.

## 2. Methods

A literature search was performed on PubMed with the keywords “fungal infections in the Caribbean”, which yielded 351 results. The earliest publication was in 1952 and the latest was in 2023. There were no classical articles, clinical trials, books, or documents that were found in the search. The majority of the results were case reports about fungal infections but not necessarily related to the Caribbean. There were clinical studies, review articles, and systematic reviews that composed the rest of the search results. The inclusion criteria included any piece of literature that was relevant to a fungal infection in the geographical region of the Caribbean, and the literature pieces that did not talk about fungal infections or were not based on infections in the Caribbean were excluded. This resulted in a total of 37 articles, which included letters to the editor, correspondence, letters, original articles, review articles, case reports, and clinical studies.

## 3. Results and Discussion

After analyzing the literature available for review, we found it best to characterize the information available to us in terms of fungal burden in major countries and regions of the Caribbean with relevant overlaps wherever required. The incidence and prevalence of fungal diseases varied widely across the region, with some diseases being more prevalent in certain countries. For example, histoplasmosis was found to be highly prevalent in the Dominican Republic, while cryptococcosis was more common in Jamaica. Several studies reported an increase in the incidence and/or prevalence of fungal diseases over time. 

### 3.1. Trinidad and Tobago 

Data on the incidence and prevalence of serious fungal diseases in Trinidad and Tobago (TT) are limited. However, several studies have reported the prevalence of specific fungal infections in the country. A few factors that may have led to this could be a lack of general awareness, a lack of infectious disease experts to diagnose patients, a lack of laboratory facilities to perform specific tests, and no interest from local health departments in fungal diseases even though some of these infections are deadly.

There have been reports of tinea capitis in children, histoplasmosis, paraccocidioidomycosis, and mycetoma, but there were no data available on the morbidity and mortality of these diseases. This changed when Denning and Gugnani in 2015 estimated the burden of serious fungal infections in the country. The population of TT at the time of the study was 1.339 million people [4]. Since not many published data existed at the time of their study, they used whatever local data were available along with the literature estimates of the incidence and prevalence of fungal infections and found an estimated 31,000 people to be suffering from fungal infections in TT each year (these are not validated using local epidemiological studies) [4]. The authors mentioned in their review that histoplasmosis is relatively unknown by the public in the Caribbean and that TT has the ideal environmental factors for it. Asthma is relatively common on the island, and they estimated that the incidence of ABPA would be high, given that the people with asthma who do not improve after conventional treatments do well after antifungal therapy [4]. They did not investigate data about cutaneous infections in TT given that these are less serious. The rates of invasive fungemia were also estimated, but the authors stated that it is likely that the number is much higher than their estimate given the lack of generally used blood cultures. The authors strongly recommended the creation of more public health awareness and the establishment of a good laboratory in TT with the capacity of testing for fungal infections [4].

This initial review of fungal infections in TT led to another extensive study that was performed by Edwards et al., in 2021 [5], where the authors set out to re-estimate the incidence and prevalence of serious fungal diseases in TT. They searched the medical literature extensively for published data, and, if data were unavailable, they used the frequency of infections in the populations at risk to obtain logical estimates. For example, they used asthma and pulmonary tuberculosis rates to estimate the prevalence of ABPA. The population of TT is now 1.395 million. The authors estimated a fungal burden of 46,156 individuals, 3.3% of the population. This included 21,455 women with recurrent vulvovaginal candidiasis, 4800 adults with SAFS, 3637 people with ABPA, 178 people with chronic pulmonary aspergillosis, and an additional 115 with invasive aspergillosis. They also researched cutaneous mycoses, found about 14,647 cases of tinea capitis in children, and estimated a candidemia rate of around 70 cases/year. There were 11,000 people living with HIV-AIDS, and, amongst these people, they estimated 124 cases of *Pneumocystis jirovecii* pneumonia, 88 cases of disseminated histoplasmosis, and 40 cases of cryptococcal meningitis [5]. Prior to 2021, no cases of ABPA in TT had been reported in the literature. Dalip D et al. (2021) presented the first documented case of ABPA in TT with the aim of increasing awareness about its diagnosis and management in the region [6]. A 73-year-old female presented with a persistent, non-productive, nocturnal cough with associated wheeze, dyspepsia, allergic rhinitis, and significant weight loss over six months. The clinical findings included fine crepitations in the right lung bases, an elevated eosinophil count, and high IgE levels. On imaging, a chest X-ray revealed right basilar infiltrates, while high-resolution computed tomography (HRCT) showed specific changes in the right middle lobe, indicative of ABPA. Pulmonary function tests demonstrated a restrictive defect, and the diagnosis of ABPA was confirmed through a positive *Aspergillus* IgE antibody test, along with peak flow diary readings which indicated asthma variability. This patient was optimized with oral prednisone and anticholinergics. This case, overall, highlights the need for awareness and early diagnosis of ABPA in Trinidad and contributes valuable information for its proper management.

Another recent study published by Edwards et al. looked at the incidence of histoplasmosis and cryptococcal antigenemia among patients who attend a large HIV clinic in TT. Histoplasmosis and cryptococcosis are serious co-infections in people with HIV, causing severe illnesses and high mortality rates [7]. The study aimed to determine the incidence of these infections and evaluate diagnostic methods. The researchers used antigen detection tests for diagnosis due to limited laboratory resources. Among 280 patients with CD4+ counts below 350 cells/mm³, 6.4% were diagnosed with probable disseminated histoplasmosis, and 2.5% had cryptococcal antigenemia. The researchers suggested integrating antigen tests into clinical laboratories for quicker diagnosis and treatment access [7].

### 3.2. Jamaica

Jamaica is one of the largest countries in the Caribbean, with a population of about 2.9 million people. Just like TT, the fungal burden in Jamaica is still largely unknown, but the article by Denning et al. [8] sheds some light on fungal infections in the country. The authors searched for the available medical literature, and, in the instances where it was not available, they used similar estimation methods. The results of the study showed that around 57,600 people suffer from fungal infections each year [8]. Jamaica is estimated to have 5116 ABPA cases and 6753 SAFS cases. About 42,885 women experience recurrent vulvovaginal candida infections yearly. The HIV prevalence was around 27,000 patients in 2013, now 30,000, and, using conservative estimation rates, the authors expect between 2100 and 6300 fungal infections in these patients, alongside 1120 cases of *P. jirovecii* pneumonia and 140 cases of cryptococcal meningitis. They were unable to estimate the burden of invasive aspergillosis, histoplasmosis, and mycetoma as the data available were so scarce. Tinea capitis is common in children, but also not estimated. A retrospective study was conducted by East-Innis et al., which aimed to identify the predominant dermatophyte fungi causing tinea capitis in Jamaica. It analyzed fungal culture requests at the University Hospital of the West Indies from 1998 to 2002. The results indicated a shift from *Microsporum audouinii* (61.5%) in 1998 to *Trichophyton tonsurans* (85%) in 2002 as the predominant species. The average age was 8.6, with females comprising 55.7% of the positive cases [9]. Data on the burden of histoplasmosis in Jamaica are scarce, but evidence suggests its presence. A case study by A. Fincham found that 25 out of 28 cavers had originally developed a cough and fever after visiting the wet St. Clair cave [10]. They were all found to be positive for histoplasmin. This challenged the conventional wisdom that histoplasmosis is linked only to dusty caves and suggests that the inhalation of spores in bat excreta can also pose a risk in wet caves.

### 3.3. Dominican Republic 

The Dominican Republic (DR), in combination with Haiti, accounts for approximately 75% of HIV infections in the Caribbean region [11]. Histoplasmosis is endemic in the DR. However, there is an alarming scarcity of data within the literature attesting to the prevalence of fungal infections in those residing in the DR. This can be attributed to several factors, which were brought to light during a devastating outbreak of *Histoplasma capsulatum* in tunnel workers in 2015 [12]. Upon further investigation by the US Centers for Disease Control and Prevention, 83% of the 36 male tunnel workers (average age 32 years and previously healthy) met the case definition for severe acute histoplasmosis infection. This outbreak resulted in 93% hospitalizations, 30% intensive care admissions, and 10% deaths. This severe outbreak and deaths in these workers were linked to a general unfamiliarity with the disease, leading to a delayed diagnosis and, subsequently, late treatment.

Similarly, there is a scarcity of data on fungal infections within the DR. Gugnani et al. estimated the burden of serious fungal diseases using 2014 reports by the World Health Organization Stop Tuberculosis program and the Joint United Nations Program on HIV/AIDS (UNAIDS) [11]. They estimated that, of the 10.09 million population, approximately 221,027 (2%) had a serious fungal infection. This includes an estimated 158,134 women with relapsing vulvovaginal candidiasis, approximately 34,000 persons with SAFS, and 25,150 persons with ABPA. Chronic pulmonary aspergillosis was also estimated to have an annual prevalence of 1374 cases, often linked to pulmonary tuberculosis [11]. Histoplasmosis, fungal keratitis, and tinea capitis could not be estimated.

Other studies have been attempted to track the prevalence of superficial fungal infections. In a study of school-aged DR children with tinea capitis, *T. tonsurans* was the leading cause (61.16%), followed by *M. audouinii* (24.27%) and *M. canis* (11.65%) [13]. While there are a concentrated number of reports of chromoblastomycosis infections within the subtropical and tropical regions, specifically located between 30° N and 30° S, there is no documented incidence or prevalence of these infections within the DR [14]. Dermoscopy is a tool that can be used to diagnose tinea capitis, a common fungal infection in children, diagnosed through mycological examination. The research conducted by Arrazola-Guerrero et al. aimed to describe dermoscopic patterns in patients with tinea capitis. They conducted a study on 37 patients, observing patterns like “comma hairs”, “corkscrew hairs”, short hairs, and black dots. Other findings included scales, peripilar casts, alopecia, pustules, and meliceric crusts [15]. The study highlights the potential of dermoscopy as a diagnostic tool when microscopes or mycology labs are inaccessible. However, there is an urgent need to generate more data measuring the true rates of fungal infections.

Chromoblastomycosis, another common dermatological fungal infection in the tropics, is a chronic, progressive fungal infection characterized by melanized fungi, primarily affecting those with prolonged contact to soil [14,16,17]. In a review of Latin America by Guevara et al., the countries with the highest reported cases of chromoblastomycosis included Brazil (37.1%), followed by Venezuela (14.4%), Mexico (14; 10.6%), and Cuba (9.8%) [16]. Interestingly, many of the cases reported dermatologic lesions and ulcerations on the lower limbs; however, in those residing in Cuba and in the rare cases from the DR, the found lesions were primarily located on the upper limbs. This could be attributed to an underreporting of the disease, as reporting chromoblastomycosis is not mandatory [16].

Common causative agents of chromoblastomycosis include *F. pedrosoi* (84.1%), with *C. carrioni* (11.6%) and *Phialophora verrucosa* (1.1%) being less commonly isolated [18]. Isolates of the *Fonsecaea* species made up approximately 80% of Cuban reports, as well as approximately 66% of the three reports available from the DR [10,16]. Long-term chromoblastomycosis infections are associated with the development of squamous cell carcinoma and melanoma; therefore, early medical detection and treatment are crucial to reducing or lowering a future disease burden on the patient and their community [14,16,17,18].

### 3.4. Haiti

Similar to other Caribbean regions, there is a discordance between the endemic mycoses of the Caribbean and the sparsity of fungal infection reports in Haiti. Haiti is one of the poorest nations within the Western hemisphere [19]. Haiti has suffered significant natural disasters including hurricanes, floods, and earthquakes that have led to numerous global efforts towards increasing medical support for its inhabitants (borda). Despite all these efforts, poor access to medical care, disease diagnosis, and lack of treatment modalities run rampant, all of which contribute to the apparent low prevalence of fungal infections in this country [19]. There are currently about 140,000 people with HIV (including 6500 children) in Haiti, with more women affected than men and nearly twice as many as in the DR as per the 2022 UN HIV and AIDS estimates. Haiti was a big focus of research activity early in the HIV outbreak, but the last publication on life-threatening fungal diseases was in 2000.

Among young women with STIs, 9% had *Candida* detected [20]. There was an absence of reports of any tinea capitis in Haiti until 1988, when a prospective study in Port-au-Prince revealed 64 children with tinea capitis [21]. This study recognized five major dermatophytes as the major contributors in this outbreak. *T. tonsurans* accounted for approximately 63.6% of all the cases, followed by *T. mentagrophytes* (14.5%), *M. audouinii* (12.7%), *T. rubrum* (7.3%), and *M. gypseum* (1.8%) [21]. The study suggests that the sudden increase in tinea capitis cases may be due to holiday travel and frequent immigration and emigration between Haitians and residents of the United States. Since *T. tonsurans* is particularly contagious, this theory is not an unreasonable one. However, it could be argued that recent increases in testing the pediatric population for tinea capitus could have simply unmasked an ongoing issue under the guise of a sudden, increased prevalence. Regardless, untreated dermatophyte infections in Haiti are an emerging problem affecting the pediatric population of Haiti.

One additional study investigated the prevalence of dermatologic infections in those that live in urban Haitian areas. They revealed that 40% of the population suffered from a skin infection, with fungal infections accounting for 21.9% of all skin infections, with a majority due to infections of the *Malassezia* species and dermatophytes [19]. While there is an alarming lack of fungal infections reported in Haiti, the limited reports that are available suggest that fungal infections, particularly those in the pediatric population, need better intervention to prevent their continued spread. 

A Case Series by Hulin et. al explores mycetoma, a neglected tropical disease, in Haitian migrants. The cases presented involve a 53-year-old female farmer who underwent a trans-tibial amputation due to advanced fungal mycetoma caused by *Madurella mycetomatis* and a 35-year-old pregnant woman with bacterial mycetoma treated successfully with cotrimoxazole. Despite Haiti being considered non-endemic, the study suggests endemicity for both fungal and bacterial mycetoma, urging increased awareness among dermatologists. The authors stress the need for further prevalence studies in Haiti and the Caribbean, emphasizing early detection to prevent severe outcomes such as amputation [22].

Treating fungal infections in Haiti can be challenging due to the high cost of antifungal medications and the lack of trained healthcare professionals. Many people in rural areas rely on traditional medicine or self-medication, which can lead to ineffective treatment and the spread of drug-resistant strains of fungi. To address this issue, organizations like the Haitian Society of Mycology and the Pan American Health Organization (PAHO) are working to raise awareness about fungal infections and improve access to affordable and effective antifungal treatments.

### 3.5. Cuba

The humid and warm climate, poor hygiene, and inadequate sanitary conditions contribute to the prevalence of fungal infections in Cuba. After the first reported case of HIV from Cuba in 1986, reports of *Cryptococcus* infections increased to 8–15 patients annually [23]. Earlier cryptococcal infection was only seen in patients with severe alcoholism, organ transplants, and genetic immunodeficiencies. In a study of 211 serial autopsies in patients with AIDS, a staggering 29% had evidence of severe systemic or central nervous system infections with the *Cryptococcus* species [23,24].

While environmental niches for *Cryptococcus neoformans* and *Cryptococcus gattii* complex within Cuba have yet to be identified [23,25], it is presumed that these infections are generally acquired during childhood. This is attributed to a high prevalence of positive serologic studies indicating a previous infection with *Cryptococcus* at a young age in the Cuban population [23]. This is supported by a recent study of Cuban children with Cryptococcal infections [23,26]. None of the children with Cryptococcal infections in Cuba were HIV positive or had other underlying immunosuppressive conditions [26]. In a review of 97 Cuban patients treated at the Tropical Medicine Institute ‘Pedro Kouri’ who were diagnosed with *C. neoformans*, 84.5% had preexisting HIV infections, while the remaining 13.5% were immunocompetent [26]. Apparently, immunocompetent patients may have a severe infection, but suspicion of cryptococcal disease may be low, meaning that testing for this mycotic infection has not been carried out [23].

A significant proportion of opportunistic infections can also be attributed to *P. jirovecii,* especially among immunocompromised patients [27]. The identification of *P. jirovecii* colonization in asymptomatic patients living in endemic areas, such as Cuba, can greatly aid in determining its transmission and epidemiology [27]. This is essential as there is still a lot of uncertainty surrounding the transmission and epidemiology of *P. jirovecii* in Cuba [28]. Colonization with *P. jirovecii* has been reported to increase to risk of *P. jirovecii* infections in adults and was linked to the sudden infant death syndrome, as well as bronchiolitis in infants and children [29].

The mitochondrial small subunit (mtSSU) rRNA and the mitochondrial large subunit (mtLSU) rRNA have been studied to help characterize the rampant *P. jirovecii* strains in Cuba [28]. Multilocus genotyping can be useful to track the fungal strain typing and geological diversity and determine fungal burden in the scope of patient disease prognosis and clinical outcome. One study by de Armas et al. identified three genotypes of mtSSU rRNA. Genotype I (85C/248C) of the mtLSU rRNA gene was found in only 12.5% of Cuban patients, but was highly common in Spain (64%), Portugal (49%), and the USA (38%). Genotype II (85A/248C) was mostly found in French and American patients and was rarely seen in the Cuban population [28]. Interestingly, Genotype III (85T/248C) was found in 81.3% of Cuban patients and was rarely seen in French patients. Similar results have been previously reported [16]. This particular strain of *P. jirovecii* is probably linked to Cuba being an island with limited international contact, where a limited number of strains are repeatedly transmitted to its own inhabitants.

As *P. jirovecii* cannot be cultivated in vitro, the determination of antimicrobial sensitivity and resistance using traditional assays is impossible [29]. However, the analyzation of the dihydropteroate synthase (DHPS) loci, the major target of sulfonamides, can potentially identify strains of *P. jirovecii* resistant to these agents. In a study by Ernesto et al., an RFLP analysis of DHPS PCR products sampled from Cuban infants revealed four separate genotypes of *P. jirovecii*, of which 18% were resistant to sulfonamides [29].

Chromoblastomycosis usually begins with the introduction of conidia or hyphae through skin trauma or breaks [16,17]. Interestingly, patients might not recall traumatic events, leading to delayed medical attention and unnoticed disease progression [16]. Chromoblastomycosis constitutes roughly 9.8% of fungal infections, predominantly linked to *F. pedrosoi* [17,18]. Rare cases in Cuba involving *F. monophora* were identified through ITS rDNA sequencing [18]. These cases exhibited slow lesion growth over years and were treated with extended topical antifungal therapy. Surgical excision became necessary when the topical treatment showed a limited efficacy, eventually leading to the successful clearance of the infection after years of follow-up. The challenging nature of chromoblastomycosis therapy is evident in the mentioned cases. Due to its chronicity, treatment difficulty arises, often leading to relapses and up to 85% medication failure rates [2,18]. The diverse fungal agents causing chromoblastomycosis and the lack of established therapeutic norms contribute to this complexity. The primary oral antifungals employed include itraconazole, terbinafine, and 5-flucytosine, usually administered in a combination for 6–12 months [2]. Although newer options like posaconazole show promise, cost remains a barrier. While topical antifungals are an option, they exhibit a higher failure rate [2]. In regions with limited access to antifungal drugs or surgery and reduced patient follow-up, chronic cases of chromoblastomycosis can prevail. Chronic pulmonary aspergillosis (CPA) is a severe fungal infection primarily affecting individuals with underlying respiratory conditions. Rodriguez et al. conducted a case series involving 47 patients of the Benéfico-Jurídico Pneumological Hospital [30]. Among these patients, 21 were diagnosed with CPA based on clinical signs, radiological evidence, mycological culture, and *Aspergillus* IgG antibody analysis. The study also investigated patients with elevated *Aspergillus* IgG antibodies and the presence of cavities, examining how frequently they were misdiagnosed due to their resemblance to other respiratory conditions, such as tuberculosis. The diagnosis of CPA requires a comprehensive approach involving clinical assessment, radiological imaging, and laboratory tests. Overall, the above-mentioned research contributes valuable information to the field of aspergillosis, with implications for improved diagnosis and management strategies. 

### 3.6. Martinique/French West Indies (FWI)

Fungal infections are a common health issue in Martinique, a French Caribbean Island located in the Lesser Antilles. The tropical climate, high humidity, and abundant rainfall make it an ideal environment for fungi to thrive. The annual incidence of disseminated histoplasmosis of Martinique is similar to that of the United States (0.24/100,000) [31]. There are isolated reports of histoplasmosis in residents of Martinique. In a review of unpublished data, ten patients, nine males and one female, residing within the French West Indies (FWI) had confirmed cases of histoplasmosis between 1991 and 1997. Of these ten patients, eight presented with dermatologic manifestations of histoplasmosis, which is generally considered unusual. Of the ten patients, two had cutaneous nodules; two had molluscum contagiosum-like lesions; two had skin ulcerations; one had violaceous papules of the arms, and one had several erythematous squamous lesions of the abdomen [31]. Recent epidemiological investigations of disseminated histoplasmosis have reported high positivity rates in the population of Martinique (12%), which is comparable to other Caribbean islands, such as Cuba, with 13–28.8% [31,32], Trinidad, with 42%, Guyana, with 29%, and Barbados, with 4% [31,33].

The study by Agossou et al. reports four cases of Histoplasmosis in individuals without known immunocompromising conditions.Histoplasmosis, is typically observed in immunocompromised individuals or those with substantial exposure to the fungus. The Caribbean, including the French West Indies and French Guiana, has reported cases of histoplasmosis.

The presented cases involve a journalist and snake/mice hobbyist, a carpenter, a market gardener with potential bat droppings exposure, and a male with uncertain exposure. Despite lacking clear exposure history, all cases presented with acute or subacute febrile dyspnea. Diagnosis relied on various methods such as histological analysis, mycological culture, PCR, and serology. Treatment with antifungal medications, including liposomal amphotericin B and itraconazole, resulted in favourable outcomes in all cases [34].

These findings highlight the significance of considering acute pulmonary histoplasmosis in the differential diagnosis, particularly in endemic regions. The study emphasizes that this condition can occur in immunocompetent individuals without obvious exposure, underscoring the need for increased awareness and vigilance in clinical practice.

Fungal keratitis, a severe eye infection, poses a significant threat to vision. Although reports of fungal eye infections are very rare in the FWI, there was a significant isolated outbreak of keratomycosis attributable to *Fusarium solani* in contact lens wearers in 2005–2006 [35]. This large global outbreak was linked to a specific brand of contact lens, with corneal abscesses ranging from 2 mm to larger than 5 mm in size. This outbreak was most notably reported in Martinique, the United States, Singapore, and Hong Kong. Fortunately, there was a reasonably favorable outcome with retained visual acuity in 35.7% of the cases, with leukoma and full clinical recovery presenting in an additional 28.6% [35]. However, a penetrating keratoplasty was necessary in 35.7% of the patients, with two recurrences after transplantation. Challenges in the management of fungal keratitis include delayed patient presentation, inadequate initial treatment, and use of topical corticosteroids that worsen fungal keratitis. In a systematic review and meta-analysis conducted by Li & Denning in 2023, corticosteroids were found to alter the pattern of fungal growth, promoting more vertical penetration of the cornea. In cases requiring corneal grafting, which carries a risk of rejection, a separate study reported lower treatment failure rates when corticosteroids were used post-surgery to prevent graft rejection. This study also noted a low recurrence rate of fungal keratitis in the corticosteroid-treated group. These findings underscore the complex relationship between corticosteroids and outcomes in fungal keratitis, suggesting caution in their use and highlighting potential benefits in specific post-surgical scenarios [36]. Surprisingly, antifungal eye drops, despite being on the WHO essential medicines list, remain inaccessible in the Caribbean. Diagnostic capabilities (mainly microscopy and culture) and expertise in identifying fungal keratitis are often lacking in many regions. Adding to the concern, the last WHO guidelines (from Southeast Asia) for managing this condition date back to 2004. Fungal keratitis affects more than a million people yearly, with a substantial risk of eye or vision loss. A recent estimate of the annual incidence of fungal keratitis in the Caribbean suggested 1305 cases [37].

### 3.7. Guadeloupe

Not much information exists about fungal infections in Guadeloupe but there is one documented case report about a patient with mucormycosis, which was managed with medical as well as surgical treatment [38].

### 3.8. Cooperative Republic of Guyana (Guyana)

Like the outbreak of histoplasmosis in the DR, an isolated and devastating outbreak of histoplasmosis was also reported in Guyana in 2019 [39]. Guyana is in the Guinea Shield, notorious for high rates of histoplasmosis, documented in the almost-neighboring French Guiana as well. Fifteen previously healthy Chinese migrant workers were hired to work in a manganese mine in Guyana. Soon after the initiation of work, six reported symptoms of respiratory infection with cough, weakness, headaches, and joint pain. These workers required intensive care, and the infection eventually resulted in the death of two of the workers shortly after initial presentation [39]. Further investigations of the outbreak revealed that the tunnels had been abandoned for 50 years and bats were living inside, with abundant bat guano throughout the tunnels. Additionally, the workers did not have proper personal protection equipment. Since the close contacts of the patients, who did not enter the tunnels, remained asymptomatic and tested negative for histoplasmosis, the theory of person-to-person transmission is highly unlikely. This suggests that the pathogenic agent was within the tunnels. Overall, this outbreak resulted in an attack rate of 38.5%, with an exposure’s odds ratio of 2.4 (*p* < 0.0001) [39]. *H. capsulatum* is found in the soil and in bird/bat droppings. This outbreak highlights the importance of spreading awareness of fungal infections in areas where they are more likely to be prevalent, such as in warm and humid environments of the Caribbean region. An estimate of the burden of disseminated histoplasmosis in Guyana, using 2012 HIV data and the literature up to 2015, found a range of annual incidence of 61–143 cases, in comparison with 200 cases of tuberculosis [40]. There are no reports of any other fungal infection in Guyana. 

**Figure 1:**
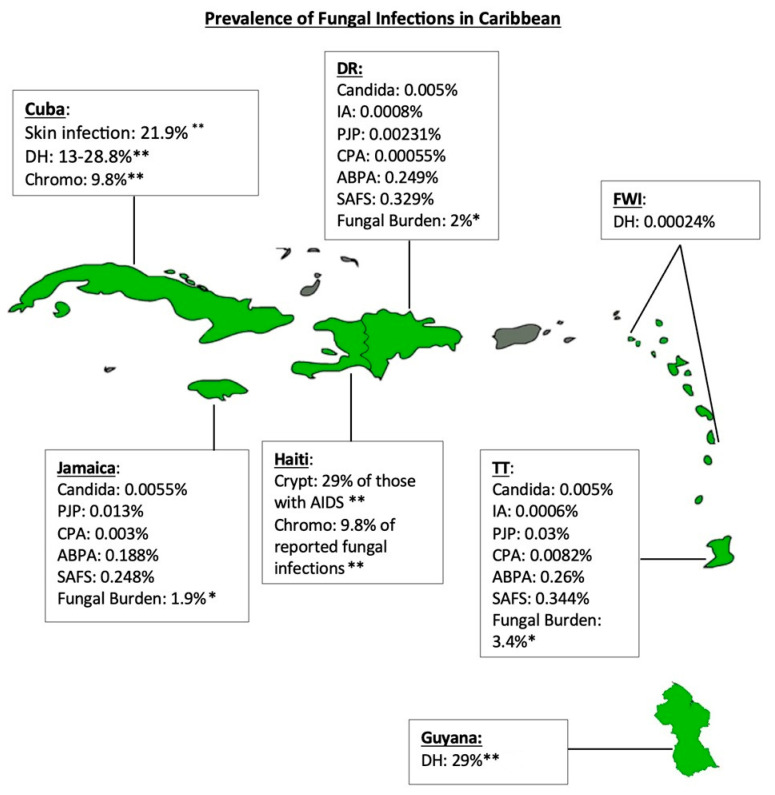
Fungal Infection Prevalence in the Caribbean: map of the Caribbean countries discussed (green) and the available data regarding the reported and estimated prevalence of fungal infections per country based on the literature currently available. Key: DH: disseminated histoplasmosis; Chromo: chromoblastomycosis; PJP: Pneumocystis jirovecii pneumonia; CPA: Chronic pulmonary aspergillosis; SAFS: Severe asthma with fungal sensitization; ABPA: Allergic bronchopulmonary aspergillosis; IA: Invasive aspergillosis; Crypt: Cryptococcus infections. * Percentage of the entire population with a fungal infection; and ** percentage of specific, reported fungal infections.

Figure 1 Depicts the map of the Caribbean countries and the available data regarding the reported and estimated prevalence of fungal infections per country.

## 4. Diagnostic Capability

A survey conducted by Falci et al. (2019) assessed the diagnostic and therapeutic capabilities in the field of clinical mycology in Latin America and the Caribbean. The survey involved 129 centers in 24 countries and aimed to identify the current state of diagnostic tools, the availability of antifungal drugs, and the need for improvement in the region. The findings reveal that only 9% of the centers met the minimum mycology standards [41].

The Caribbean is hyperendemic for histoplasmosis. Early detection of this disease is quintessential in this high-risk population. Traditional diagnostic methods like microscopy and histopathology have limitations in sensitivity and specificity [42]. Laboratory cultures, though considered the gold standard, are slow and not always feasible due to safety requirements. Antibody detection is not widely available. The introduction of antigen detection tests, which are quick and reliable, has revolutionized diagnosis, especially for disseminated histoplasmosis. PCR-based approaches also hold promise [42]. A recent article by Caceres et al. in 2019, more commonly known as the Manus Declaration, investigated the current situation of histoplasmosis in the region. Only three countries in the Caribbean region were mentioned in the study—Guyana, Jamaica, and the Dominican Republic. The data showed there were no antigen tests or molecular tests available at any laboratories in Guyana and Jamaica, whereas only the antigen test was available in the Dominican Republic [43]. The Manaus Declaration set a goal to ensure that every country in the Americas has access to swift histoplasmosis testing (either through antigen or molecular methods), as well as itraconazole and lipid-based forms of amphotericin B, by the year 2025, a target referred to as “100 by 25” [43].

The aim should not just be limited to histoplasmosis, but a diagnostic portfolio is needed in the Caribbean region for fungal infections in general, to reduce patient morbidity and mortality, including monitoring patient outcomes after receiving appropriate therapy. However, due to the limited availability of laboratory diagnostic materials and a shortage of healthcare workers with the expertise to interpret diagnostic results, there are very limited data on fungal infections in this region [5,40]. A modern mycology laboratory should have the tests depicted in Table 1 below, as described by the Global Action for Fungal Infections (GAFFI) [44,45]. While there are still many other methods of diagnosis of fungal infections, such as latex agglutination tests and other such lateral tests, the GAFFI recommends that, at a minimum, a mycology laboratory should have the diagnostic testing methods listed in Table 1 [45].

In addition, availability of antifungal agents is also an important issue. Fluconazole, itraconazole, voriconazole, amphotericin B, flucytosine, and echinocandins (including micafungin, caspofungin, and anidulafungin), along with natamycin eye drops, are included in the World Health Organization’s Essential Medicines List [45,46]. As per the data available at the GAFFI, most of the countries in the Caribbean have some availability of the above-mentioned drugs, but no information is available about how accessible they are in clinical practice (Table 2) [45,47]. Additionally, there are no reported data describing the outcomes of patients with fungal infections who received antifungal treatment.

The WHO accepted rapid tests for life-threatening fungal diseases aspergillosis and Pneumocystis pneumonia as part of its essential diagnostics in 2021. There should be consideration given to histoplasmosis as well, especially given how endemic the region is for the disease and the complications it causes to immunocompromised patients.

The dynamic interplay between climate change and fungal infections underscores a critical facet of emerging health challenges. The evolving environmental landscape, shaped by climate change, has profound implications for the prevalence and nature of fungal infections in human populations [48]. Notably, alterations in temperature, precipitation patterns, and other environmental parameters contribute to the expansion of the geographic footprint of endemic fungi, introducing novel pathogens which may pose heightened risks of infections. Furthermore, the changing environment is implicated in elevating the susceptibility to fungal infections among individuals, accentuated by factors such as increased population density, urbanization, and the prevalence of viral infections like COVID-19 and severe influenza. Additionally, climate-driven selective pressures may instigate the development of drug-resistant strains, amplifying concerns regarding the efficacy of antifungal treatments [48]. A comprehensive understanding of these multifaceted impacts is essential for devising effective strategies to manage and prevent fungal diseases in the context of an evolving environment.

Urgent attention is required to enhance diagnostics, access safer antifungal drugs, and develop guidelines for the use of antifungal drugs and diagnostic methods via medical societies. This review highlights the scarcity of diagnostics and therapy despite prevalent mycoses. Addressing these issues is crucial to bridging regional gaps and elevating capabilities in the region.

Table 3 summarizes the publications regarding serious fungal diseases in the Caribbean till date and full details are given in Supplementary Table S1.

## 5. Conclusions

Fungal diseases are an important public health problem in the Caribbean. The epidemiology of these diseases varies widely across the region, highlighting the need for targeted interventions and surveillance. This review highlights the current data available in addition to highlighting where more efforts are needed to improve patient outcome. The high burden of fungal diseases in the Caribbean is likely due to a combination of factors, including environmental factors, socioeconomic factors, and the high prevalence of risk factors such as HIV/AIDS and diabetes. However, there are still significant gaps in the available literature, which impede a complete understanding of the fungal burden within this region. Implementing national surveillance systems and improving access to diagnostic testing could provide further insight into this issue. Further research is needed to better understand the epidemiology of fungal diseases in the Caribbean and develop effective prevention and treatment strategies.

## Figures and Tables

**Table 1:** Core diagnostic tests for a modern mycology laboratory (adapted from the 2015 Roadmap of GAFFI).

Diagnostic Tests Recommended by the GAFFI [44]		
Test	Infection	Diagnostic Sensitivity
Direct microscopy	Invasive infections, skin, hair, and nails, VVC	30–90%
Antigen	Cryptococcal meningitis	99%
PCR on respiratory samples	*Pneumocystis* pneumonia	98%
Antigen (ELISA) on serum and respiratory samples	Invasive aspergillosis	80%
Glucan detection	Most fungal infections, high NPV allowing therapy to be stopped	65–77%
Aspergillus IgG antibody	Chronic pulmonary aspergillosis	80–95%
Aspergillus IgE	Screen for ABPA in asthma	>95%
Fungal culture and identification	All except *Pneumocystis*	10–50%
Molecular identification from histopathology positive, culture negative	All, especially mold infections	50–60%
Itraconazole and voriconazole blood levels. TDM	Aspergillosis	100%

ABPA—allergic bronchopulmonary aspergillosis; NPV—negative predictive value; TDM—therapeutic drug monitoring; VVC—vulvovaginal Candidiasis.

**Table 2:** WHO Essential Antifungal Drugs and their availability (adapted from the antifungal drug maps from the global action against fungal infections) [45,47].

Key Antifungal Drug Availability by Country [44,45]																
	Fluconazole				Itraconazole				Amphotericin B				Flucytosine			
Name	Available	Registered	EM	Formulations	Available	Registered	EM	Formulations	Available	Registered	EM	Formulations	Available	Registered	EM	Formulations
Cuba	available	registered	Y	6	available	registered	Y	1	available	registered	Y	3	available	available	Y	1
Dominican Republic	available	registered	No Data	7	available	registered	N	3	available	registered	Y	2	Not available	Not registered	N	0
Guadeloupe	available	No Data	No Data	0	available	No Data	No Data	0	available	registered	No Data	0	Not available	No Data	No Data	1
Guyana	available	No Data	No Data	0	available	registered	No Data	0	Not available	Not registered	No Data	0	Not available	No Data	No Data	0
Haiti	available	registered	Y	0	available	Not registered	N	1	available	registered	N	0	Not available	No Data	No Data	0
Jamaica	available	registered	Y	3	available	registered	N	1	available	registered	Y	0	Not available	No Data	No Data	0
Martinique	available	No Data	No Data	0	available	registered	Y	1	available	registered	No Data	0	Not available	No Data	No Data	0
Trinidad and Tobago	available	registered	No Data	1	available	registered	No Data	0	available	registered	No Data	0	Not available	No Data	No Data	0
Cuba	Not available	No Data	N	0	Not available	No Data	N	0	available	No Data	Y	1	Not available	No Data	N	0
Dominican Republic	available	No Data	Y	1	available	No Data	No Data	4	available	No Data	N	1	Not available	No Data	N	0
Guadeloupe	available	No Data	No Data	0	available	No Data	No Data	0	Not available	No Data	No Data	0	available	No Data	No Data	1
Guyana	available	No Data	No Data	0	Not available	No Data	No Data	0	Not available	No Data	No Data	0	available	No Data	No Data	1
Haiti	available	No Data	No Data	0	available	Not No Data	No Data	0	Not available	Not No Data	No Data	0	available	No Data	No Data	1
Jamaica	available	No Data	No Data	0	available	No Data	No Data	0	Not available	Not No Data	No Data	0	available	No Data	No Data	1
Martinique	available	No Data	No Data	0	available	No Data	No Data	0	Not available	No Data	No Data	0	available	No Data	No Data	1
Trinidad and Tobago	available	No Data	No Data	0	available	No Data	No Data	0	Not available	No Data	No Data	0	available	No Data	No Data	1

Registration and availability of WHO essential antifungal medications; EM, Essential Medication; Y, yes; N, No.

**Table 3:** Summary of publications regarding serious fungal diseases in the Caribbean (full details given in Supplementary Table S1).

Country	Publication Year	Fungal Disease	Patients Studied	Reference
Trinidad and Tobago	2015	Serious fungal infections, prevalence estimate	-	[4]
Trinidad and Tobago	2021	Serious fungal infections, prevalence estimate	-	[5]
Trinidad and Tobago	2021	Allergic bronchopulmonary aspergillosis (ABPA)	1	[6]
Trinidad and Tobago	2023	Histoplasmosis and cryptococcosis in AIDS	280	[7]
Jamaica	1978	Histoplasmosis	308	[8]
Jamaica	2015	Serious fungal infections, prevalence estimate	-	[9]
Dominican Republic	2010	Tinea capitis in urban and rural school children	118	
Dominican Republic	2016	Serious fungal infections, prevalence estimate	-	[11]
Latin America and the Caribbean	2021	Chromoblastomycosis, integrative review of 132 articles	-	[16]
Haiti	2009	Tinea capitis in children	64	[21]
Haiti	2019	Prevalence of dermatological conditions	137	[19]
Cuba	2013	Chromoblastomycosis	2	[18]
Cuba	2014	Cryptococcosis, literature review	-	[23]
Cuba	2014	*Pneumocystis jirovecii* in infants and toddlers with whooping cough	163	[27]
Cuba	2019	Chronic pulmonary aspergillosis	47	[30]
Cuba, Spain, France	2021	Genetic characterization of *Pneumocystis jirovecii* in Pneumocystis pneumonia patients	75	[28]
Martinique	1999	Histoplasmosis	10	[31]
French West Indies	2007	Keratomycosis cases linked to *Fusarium solani* in contact lens wearers	14	[35]
Guyana	2019	Histoplasmosis among overseas Chinese workers	15	[39]
Haiti	2023	Fungal and bacterial mycetoma in migrants from Haiti: A case series	2	[22]
Martinique, Guadeloupe and French Guiana	2023	Acute pulmonary histoplasmosis of immunocompetent subjects from Martinique, Guadeloupe and French Guiana: a case series	4	[34]

## Data Availability

Data is contained within the article or Supplementary Material.

## Supplementary Materials

The following supporting information can be downloaded at: https://www.mdpi.com/article/10.3390/jof9121177/s1, Table S1: Summary of publications regarding serious fungal diseases in the Caribbean.
