# Supplementary material for: Fungal Infections in the Caribbean: A Review of the Literature to Date
Source: J Fungi (Basel). 2023 Dec 8;9(12):1177. doi: 10.3390/jof9121177 (PMC10745041; doi:10.3390/jof9121177)
Supplement: Supplementary file 1 [file jof-09-01177-s001.zip › jof-2681473-supplementary.pdf]

Supplementary Material: Table S1- Summary of publications regarding serious fungal diseases in the Caribbean

S. I	Country	Type of study	Summary	Authors (year)
1	Trinidad and Tobago	Overview with prevalence estimate	Serious fungal infections were a significant health problem, particularly among patients with HIV/AIDS and diabetes. The most common fungal infections identified were candidiasis, cryptococcal meningitis, and histoplasmosis. The prevalence of serious fungal infections in the country was estimated at about 1,024 cases per year, based on hospital discharge data. The mortality rate for serious fungal infections in T&T was found to be high, with an estimated 41% of patients with these infections dying within one year of diagnosis. Among patients with HIV/AIDS, the prevalence of serious fungal infections was particularly high, with an estimated 18% of these patients experiencing at least one such infection each year. Diabetes was also identified as a risk factor for serious fungal infections, with an estimated 60% of patients with uncontrolled diabetes experiencing at least one such infection each year.	Denning and Gugnani (2015)
2	Trinidad and Tobago	Overview with prevalence estimate	With a population of 1,394,973 million, the estimated annual burden of fungal infections in T&T was 46,156 persons, which accounted for 3.3% of the population. This included various types of fungal infections, such as recurrent vulvovaginal candidiasis in women (21,455 cases), invasive aspergillosis (118 cases), ABPA (3,637 cases), SAFS (4,800 cases), CPA (178 cases), candidemia (70 cases), tinea capitis in children (14,647 cases), and fungal infections associated with HIV/AIDS (40 cases of cryptococcal meningitis, 88 cases of disseminated histoplasmosis, and 124 cases of Pneumocystis pneumonia).	Edwards et al (2021)
3	Trinidad and Tobago	Case Report	The first documented case of ABPA in Trinidad with the aim of increasing awareness about its diagnosis and management in the region. A 73-year-old female who presented with a persistent,non-productive,nocturnal cough with associated wheeze, dyspepsia, allergic rhinitis, and significant weight loss over six months. Clinical findings included fine crepitations in the right lung bases, elevated eosinophil count and high IgE levels. On imaging Chest X-ray revealed right basilar infiltrates, while high-resolution computed tomography (HRCT) showed specific changes in the right middle lobe indicative of ABPA. Pulmonary function tests demonstrated a restrictive defect, and the diagnosis of ABPA was confirmed through a positive Aspergillus IgE antibody test, along with peak flow diary readings that indicated asthma variability. This patient was optimized with oral prednisone and anticholinergics. This case overall highlights the need for awareness and early diagnosis of ABPA in Trinidad and contributes valuable information for its proper management.	Dalip et al (2021)
4	Trinidad and Tobago	Cohort	The study aimed to assess disseminated histoplasmosis and cryptococcal antigenemia prevalence among 280 HIV patients with CD4 counts below 350 cells/mm³ in Trinidad's clinic. Using blood and urine samples, they screened for antigens with high sensitivity and specificity. Results revealed a 6.4% histoplasmosis incidence and 2.5% cryptococcal antigenemia.	Edwards et al (2023)

5	Jamaica	Literature Review with prevalence estimate	This article estimates that over 57,000 people in Jamaica are affected by serious fungal infections annually, with candidiasis, cryptococcal meningitis, and *Pneumocystis jirovecii* pneumonia being the most common. The study emphasizes the importance of early detection and effective management, particularly in individuals with compromised immune systems. Additionally, the estimated mortality rate associated with these infections is 1,023 deaths per year.	Gugnani et al (2015)
6	Jamaica	Case study	Histoplasmosis, typically linked to dusty caves, is hown to be associated with bat disturbance in wet Jamaican caves. Previously unrecorded cases of histoplasmosis in Jamaica prompted investigations. Skin sensitivity tests on 308 subjects revealed 10% positive reactions to histoplasmin, indicating prior exposure. A group of cavers in St. Clair Cave developed symptoms including coughs and fever, with 25 out of 28 individuals affected. *H. capsulatum*, the causative agent, was isolated from cave soil samples. The incident highlights the risk to cavers in tropical guano caves. Wet caves, like St. Clair, may transmit infection through inhalation of spores in bat excreta, challenging the belief that only dry caves pose a hazard. Histoplasmin conversion and non-symptomatic carriers are noted. Jamaican resident cavers showed resilience to infection.	Fincham (1978)
7	Dominican Republic	Literature Review with prevalence estimate	DR, a country with a high prevalence of HIV infection and tuberculosis. The researchers collected data from various sources, including the WHO, UNAIDS, and literature searches. They used available data on asthma and TB rates to derive estimates for CPA, ABPA, and SAFS. When specific mycosis data were not available, they used populations at risk and the frequencies of fungal infections to estimate national prevalence. The findings indicated that approximately 2.2% of the DR population (221,027 individuals) had a serious fungal infection, including a significant number of women with recurrent vulvovaginal candidiasis. The study also estimated high numbers of ABPA and SAFS cases, as well as a substantial burden of CPA. Additionally, a few cases of histoplasmosis and chromoblastomycosis were reported.	Gugnani et al (2016)
8	Dominican Republic	Cohort Study	The study found that 83% of the mining workers developed symptoms consistent with histoplasmosis, with a significant number requiring hospitalization, intensive care, intubation, and even resulting in deaths. The outbreak was attributed to exposure to a large number of *H. capsulatum* spores in an enclosed space, lack of respiratory protection, and delays in recognizing and treating the infection. The study emphasizes the need for clinician education on histoplasmosis, improved laboratory capacity for diagnosing fungal infections, and occupational health measures to protect workers from endemic fungi in the DR.	Armstrong et al (2018)
9	Dominican Republic	Cohort Study	This study investigated the epidemiology and mycological profile of tinea capitis in urban and rural areas of the DR. A total of 118 patients with tinea capitis were included, with 63 from urban schools and 55 from rural schools near the Haiti border. The majority of cases were found in boys, especially in urban areas. The most common isolated fungi in urban areas were *M. audouinii*, *T. tonsurans*, *M. canis*, and *T. violaceum*. In rural areas, *T. tonsurans* was the predominant	Arenas et al (2010)

			fungus, with *T. mentagrophytes* also present. Overall, *T. tonsurans, M. audouinii,* and *M. canis* were the most frequently identified causative organisms. The study highlights the emergence of *M. audouinii* and an increase in *T. tonsurans*, particularly in the rural border region with Haiti.	
10	Latin America and the Caribbean	Literature Review	The integrative review of 132 articles revealed the Chromoblastomycosis primarily affected male patients engaged in occupations related to plants, lumber, or soil products. The disease course varied, with an average time of 10.8 years between lesion appearance and diagnosis. The most common clinical form was verrucous (46.4%). Infections with leprosy and immunosuppressive conditions, mainly kidney transplantation and type 2 diabetes mellitus, were observed alongside chromoblastomycosis. Treatment success was reported in 63.46% of cases, with itraconazole being the most effective, either as monotherapy or in combination with other antifungals or physical methods. Early diagnosis is challenging due to polymorphic lesion characteristics, and proper surveillance and early treatment are crucial to alleviate the disease's burden on affected populations. More comprehensive epidemiological surveillance is needed to address this significant public health issue.	Guevara et al (2021)
11	Cuba	Case Report	This paper reports on two cases of chromoblastomycosis in otherwise healthy Cuban males caused by two species of melanized fungi, *F. pedrosoi* and *F. monophora.* They were identified as the causative agents based on morphological criteria and confirmed through sequencing of the internal transcribed spacer regions of rDNA.<br>In vitro, antifungal susceptibility testing of the isolates showed that, itraconazole (MIC 0.5µg/ml/0.125µg/m) and posaconazole (MIC 0.25µg/m /0.063µg/ml) had potent activity against both *F. pedrosoi* and *F. monophora.* Surgical excision was the final treatment for both patients leading to a successful cure without relapse during a follow-up of more than a year making it a viable treatment option. Newer antifungal drugs like Posaconazole due to potent *invitro* activity can be considered as an alternative treatment.	Badali et al (2013)
12	Haiti	Cross sectional study	The objective of this study was to describe the prevalence of dermatological conditions in urban Haiti, where 60% of the population resides. The study was conducted in February 2016 and included 137 patients, with the majority being women (61.3%). The mean age of the cohort was 27.7, and 75% of the participants were adults above 15 years old. The most common dermatological conditions were infectious dermatoses (40%), followed by inflammatory dermatoses (34.2%), and neurocutaneous diseases (8.4%). Fungal infections were the most prevalent within the infection group (22%), while dermatitis and acneiform skin conditions dominated the inflammatory dermatoses category. The study found that infectious skin diseases were more prevalent in men, but 40% of infectious cases affected children, indicating vulnerability in the pediatric cohort. The study had limitations, such as a small sample size, focus on urban dwellers only, and reliance on clinical diagnosis without histological or genetic confirmation. The study suggests the need to improve access to dermatological care and medication in the Haitian healthcare system, particularly antimicrobial and anti-inflammatory medications. Population education on different skin disorders is vital for better prevention. Future	Borda et al (2019)

			directions include larger-scale dermatological expeditions over an extended period to obtain more accurate epidemiological values for skin conditions in Haiti.	
13	Haiti	Cohort	In Haiti, a sharp increase of *T. tonsurans* isolates causing tinea capitis was noticed in 2005. A prospective study was conducted in Port-au-Prince from May to November 2006, involving 64 children with tinea capitis (45 males and 19 females), with an average age of 6.1 years. The study found that *T. tonsurans* was the most frequent causative agent and that children under the age of 8 years old were the most affected (>75%). The study identified five species of dermatophytes causing tinea capitis in Haitian children, *with T. tonsurans* accounting for the majority (63.6%), followed by *M. audouinii* (12.7%), *T. rubrum* (7.3%), *T. mentagrophytes* (14.5%), and *M. gypseum* (1.8%). The limited association of the Haitian population with pets, such as dogs and cats, may be a reason why *M. canis* is rarely found as a causative agent except for those in the upper class. The recent emergence of *T. tonsurans* can be linked to the increasing mobility of the Haitian diaspora and the ease of travel to more developed countries such as the USA and Canada. It has also been postulated that as Haiti borders with the Dominican Republic, *T .tonsurans* could be "locally" transferred but no published data exist on DR dermatophytoses.	Raccurt et al (2009)
14	Cuba	Review	This review aims to provide a comprehensive guide on detecting, diagnosing, and treating cryptococcosis using Cuban data and an extensive literature review. The disease's incidence increased with the emergence of AIDS and remains a major opportunistic fungal infection in HIV-infected individuals. In Cuba, cryptococcosis cases have been associated with various factors, including HIV, alcoholism, organ transplants, and immunological disorders. Clinical symptoms vary depending on the affected organ, and diagnosis can be challenging, but newer technologies like MALDI-TOF-MS offer promising options.<br><br>Cryptococcal infection treatment is challenging, with a mortality rate exceeding 20% in the first 3 months of neurocryptococcosis treatment. The recommended protocol involves an induction phase of 2 weeks followed by 8 weeks of consolidation and a maintenance phase for at least a year or based on CD4+ T cell counts. Cuban patients with cryptococcal meningitis showed the lowest treatment failures when treated with a combination of amphotericin B, 5 flucytosine, and fluconazole at the recommended dose. Isavuconazole, a new azole, displayed promising *in vitro* activity against fungal strains. Recurrent meningitis cases were attributed to different strains or reinfection rather than drug resistance. Overall, the treatment remains complex, and new azoles offer potential benefits in managing the disease. In order to improve outcomes overall there is a need for early diagnosis and treatment.	Illnait-Zaragozi et al (2014)
15	Cuba		This study looks at the occurrence of *Cryptococcus* yeast isolates in different species of trees and cacti. Previous epidemiological studies on isolates from pigeon droppings from different regions along the Cuban island showed all isolates represented *C .neoformans var grubbi* and only a few microsatellite genotypes from the environment could be linked to genotypes in humans suggesting that clinical isolates may originate from other environmental sources. In this study, 331	Illnait-Zaragozı et al (2012)

			plants were analyzed for cryptococcus yeast using conventional identification and DNA methods to identify and characterize the isolates. The most prevalent species was found to be *C.hevanenis* (36%) and approximately 33% of the isolates (n=65) could not be identified and may represent novel fungal taxa.<br>The study highlights the need for molecular confirmation for reliable identification of *Cryptococcus* isolates from the environment, as CGB (canavanine-glycine-bromothymol blue medium) positivity was found to be a poor parameter to distinguish environmental crypto isolates. AFLP analysis proved to be an efficient method for differentiating different fungal species in large collections of environmental isolates.<br>Overall, more epidemiological studies are needed to determine the exact distribution of *C. neoformans* and *C. gattii* in Cuba and to understand the ecology, evolution, and epidemiology of Cryptococcus species.	
16	Cuba	Retrospective study	This study aimed to investigate the genetic diversity of *P. jirovecii* in colonized Cuban infants and toddlers using four genetic loci: mtLSU rRNA, CYB, SOD, and β-tubulin. Multilocus genotype data (MLG) and nucleotide sequences (MLSA) were analyzed to determine the profiles and discriminatory power of each analysis. The study focused on a subset of PCR-positive nasopharyngeal swab specimens with a high *P. jirovecii* load from 2010 to 2013. Genetic methods like DNA sequencing and SSCP were used for characterization due to the lack of *in vitro* culture systems for Pneumocystis. Significant associations between specific genotypes/MLGs and patients' demographic and clinical data were not found in the statistical analysis. Further research with larger populations is needed to explore potential associations. Understanding Pneumocystis colonization could provide insights into the epidemiology and transmission of Pneumocystis pneumonia in vulnerable populations, considering temporal and geographic acquisition of *P. jirovecii*. The investigation of genotype distribution in children posed challenges due to the difficulty in obtaining relevant clinical samples and the absence of sensitive detection methods.	Monroy-Vaca et al (2014)
17	Cuba	Cohort	The research genetically characterizes *P. jirovecii* in Pneumocystis pneumonia patients from Spain, Cuba, and France using mitochondrial large subunit (mt LSU) rRNA and mitochondrial small subunit (mt SSU) rRNA. DNA from 75 patients was analyzed using PCR to detect *P. jirovecii* DNA. The mt LSU rRNA gene revealed five known polymorphisms at positions 85 and 248, with varying frequencies across locations. The mt SSU rRNA identified a new genotype (160A/196T) as the most common in the three countries, especially in Cuba (93.8%). Genotype distribution differed between countries, with genotype 3 (85T/248C) being prevalent in Cuban patients but absent in French patients. Geographic factors may influence genotype distribution, but more research is needed due to the small Cuban sample size. The study highlights the value of mt SSU rRNA genotyping in understanding *P. jirovecii* epidemiology and suggests its potential use in multilocus analysis. Further studies are recommended to explore associations between *P. jirovecii* mt SSU rRNA polymorphisms and important biological properties.	de Armas, Y et al (2021)

18	Cuba	Case series	The study conducted at the Benéfico-Jurídico Pneumological Hospital (BJPH),Cuba, involved 47 patients . Among them, 21 patients were diagnosed with CPA based on clinical signs, radiological evidence, mycological culture, and Aspergillus IgG antibody analysis. Notably, patients with elevated Aspergillus IgG antibodies were associated with cavity presence. The study emphasizes that CPA can often be misdiagnosed due to its resemblance to other respiratory conditions. Diagnosis of CPA requires a comprehensive approach involving clinical assessment, radiological imaging, and laboratory tests.<br>The study also highlights the importance of accurate diagnosis and introduction of itraconazole (Lozartil®) as the standard treatment for CPA in Cuba.	Rodríguez et al (2019)
19	Cuba	Cross sectional-prevalence	This study aimed to investigate the prevalence and genotype distribution of *P. jirovecii* in nasopharyngeal swabs from 163 Cuban infants and toddlers with whooping cough. Using qPCR and genetic analysis, the study found that 29.4% of samples tested positive *for P. jirovecii* DNA. Genotype 2 was the most prevalent (48%), followed by genotype 1 (23%), genotype 3 (19%), and mixed genotype (10%), while genotype 4 was not found in Cuba. The research also revealed high genetic variability among Cuban *P. jirovecii* strains. Factors associated with *P. jirovecii* colonization included contact with coughers and exposure to tobacco smoke. Some specimens showed DHPS mutant strains despite no prior sulfa drug exposure, indicating the presence of mutant strains in the population. The discussion emphasized geographical variation in genotype distribution, the need for monitoring mutant strains and drug resistance, and the potential reservoirs and transmission sources of the fungus.	Monroy-Vaca et al (2014)
20	Martinique	Cohort	This retrospective study investigated the epidemiology of Histoplasmosis from 1991 to 1997 in Martinique (French West Indies-FWI). Ten cases (9 male,1 female) were identified from the register of the laboratory of mycology the only laboratory to isolate and grow *H. capsulatum* in Martinique. The median age of patients identified was found to be 48 +/- 7 years. Eight of these patients had HIV, and their median CD4 lymphocyte count was low (32/mm). Skin manifestations were observed in 80% of the cases, which is considered unusually high, especially in immunocompetent individuals. Out of the 10 patients, 4 died despite treatment with amphotericin B, the preferred treatment for severe cases, while 6 patients treated with Itraconazole alone showed promising results. The incidence of histoplasmosis in the general population during this period was 0.34/100,000, whereas the incidence in AIDS patients was 1.7%, slightly lower than in endemic areas in the USA. The data confirm that histoplasmosis is endemic in the FWI, with skin involvement being a significant aspect of the disease, especially in HIV-infected individuals. Although the study had a limited number of cases, it suggests that Itraconazole could be an effective treatment option. Further research is needed to better understand and manage histoplasmosis in this region	Garsaud et al (1999)
21	Eastern Caribbean –	Overview	A preliminary survey was conducted in Barbados, Trinidad, and Guyana to assess the incidence of histoplasmosis in the general population using various methods, including histoplasmin skin tests, serology, soil isolation, and identification	Hay et al (1981)

	Barbados, Trinidad, Guyana		of cases. Of the population studied the results showed Barbados (4%), Trinidad (42%) and Guyana (29%) had a positive histoplasmin skin test. In Trinidad, once patients over the age of 60 years old were excluded the incidence increased to 69%.<br>Few histoplasmosis cases were recorded in Trinidad despite high skin test reactivity. The difference in skin reactivity between Barbados and Trinidad may be attributed to environmental factors such as soil composition. Further research is needed to understand better the epidemiology and risk factors of histoplasmosis in the Eastern Caribbean.	
22	French West Indies	Case Series	This study investigated keratomycosis cases linked to *F. solani* in contact lens wearers utilizing Bausch & Lomb ReNu with Moisture Loc between November 2005 and May 2006 at the University Hospital Center of Fort-de-France. Out of 14 recorded corneal abscess cases (8 men and 6 women, aged 17-73), most were severe (71.4%) and associated with inadequate lens care (64.3%). *F. solani* (33.3%) and *Pseudomonas aeruginosa* (25%) were identified as the main pathogens. Favorable outcomes were observed in 35.7% of cases, while 35.7% required penetrating keratoplasty with some experiencing recurrence post-transplantation. This outbreak was consistent with similar occurrences in the United States, Singapore, and Hong Kong. *F. solani* accounted for 41.6% of cases, and no other significant risk factors for keratomycosis were identified. The study highlights the increasing prevalence of keratomycosis associated with Bausch & Lomb ReNu with MoistureLoc contacts lens in the French West Indies.	Donnio et al (2007)
23	Guyana	Case study	This report discusses a cluster of infections caused by Histoplasma among overseas Chinese workers in Guyana, (2019). It is the first known occurrence of such infections in this population. The study followed 15 Chinese workers with febrile illness symptoms after working in a manganese ore mine in Guyana. Of these, 7 cases were confirmed through laboratory tests, while the others were clinically diagnosed.<br>The epidemiological investigation revealed that all infected patients worked in abandoned tunnels inhabited by bats, and their exposure intensity to the contaminated soils was strongly associated with the severity of illness. Patients with higher exposure intensity had earlier and more severe disease onset. Importantly, none of the patients used proper personal protection equipment like face masks while working, which likely increased their exposure to Histoplasma spores. This report emphasizes the need for effective education and communication initiatives among residents and travelers in these regions. However, the study was limited in assessing the environmental risk of the mines due to difficulties in transporting specimens.	Zhou et al (2019)

CPA-chronic pulmonary aspergillosis; ABPA-allergic bronchopulmonary aspergillosis; SAFS-severe asthma with fungal sensitization
